# Advanced genes expression pattern greatly contributes to divergence in *Verticillium wilt* resistance between *Gossypium barbadense* and *Gossupium hirsutum*

**DOI:** 10.3389/fpls.2022.979585

**Published:** 2022-08-01

**Authors:** Lu He, Zegang Han, Yihao Zang, Fan Dai, Jinwen Chen, Shangkun Jin, Chujun Huang, Yu Cheng, Juncheng Zhang, Biyu Xu, Guoan Qi, Yiwen Cao, Sunyi Yan, Lisha Xuan, Tianzhen Zhang, Zhanfeng Si, Yan Hu

**Affiliations:** ^1^Department of Agronomy, College of Agriculture and Biotechnology, Zhejiang University, Hangzhou, China; ^2^The Rural Development Academy, Zhejiang University, Hangzhou, China

**Keywords:** cotton, *Verticillium dahlia*, transcriptome analysis, disease resistance, defense response

## Abstract

*Verticillium*, representing one of the world’s major pathogens, causes Verticillium wilt in important woody species, ornamentals, agricultural, etc., consequently resulting in a serious decline in production and quality, especially in cotton. *Gossupium hirutum* and *Gossypium barbadense* are two kinds of widely cultivated cotton species that suffer from Verticillium wilt, while *G. barbadense* has much higher resistance toward it than *G. hirsutum*. However, the molecular mechanism regarding their divergence in Verticillium wilt resistance remains largely unknown. In the current study, *G. barbadense cv.* Hai7124 and *G. hirsutum* acc. TM-1 were compared at 0, 12, 24, 48, 72, 96, 120, and 144 h post-inoculation (hpi) utilizing high throughput RNA-Sequencing. As a result, a total of 3,549 and 4,725 differentially expressed genes (DEGs) were identified, respectively. In particular, the resistant type Hai7124 displayed an earlier and faster detection and signaling response to the *Verticillium dahliae* infection and demonstrated higher expression levels of defense-related genes over TM-1 with respect to transcription factors, plant hormone signal transduction, plant-pathogen interaction, and nucleotide-binding leucine-rich repeat (NLR) genes. This study provides new insights into the molecular mechanisms of divergence in *Verticillium wilt* resistance between *G. barbadense* and *G. hirsutum* and important candidate genes for breeding *V. dahliae* resistant cotton cultivars.

## Introduction

Due to their sessile nature, plants fatally suffer from an extensive diversity of abiotic and biotic stresses. When it comes to biotic setbacks, plants respond to pathogen infection using a two-branched innate immune system: pattern-triggered immunity (PTI) and effector-triggered immunity (ETI) (Chisholm et al., 2006; Jones and Dangl, 2006). Essentially, PTI and ETI eventually converge into many similar downstream responses, albeit with distinct amplitudes and dynamics (Yuan et al., 2021b). In recent decades, researchers have made many distinguished advances in the immunity of plants against pathogenic bacteria. Pathogen recognition by surface receptors activates multiple protein kinases and nicotinamide adenine dinucleotide phosphate (NADPH) oxidases, while intracellular receptors primarily potentiate the activation of these proteins by increasing their abundance through several mechanisms, showing immune pathways activated by cell-surface and intracellular receptors in plants mutually potentiate to activate strong defenses against pathogens (Ngou et al., 2021). Moreover, an EDS1–PAD4–ADR1 node has been proposed as a convergence point for defense signaling cascades, activated by both surface-resident and intracellular leucine-rich-repeat (LRR) receptors, in conferring pathogen immunity (Pruitt et al., 2021). It has been gradually revealed that virulent pathogens use effectors to suppress PTI as a major mechanism of pathogenesis (Yuan et al., 2021a). Consequently, mountains of genes are involved in PTI and ETI activation mechanisms. These genes work separately and crosstalk to ensure a robust immunity to detect and cope with diverse biotic attacks.

The genus *Verticillium* represents one of the world’s major pathogens, causing Verticillium wilt in many plants (Pegg et al., 2002). Of these polyphagous wilt pathogens, *Verticillium dahliae* stands out because it has the broadest host range and infects over 200 plant species (Inderbitzin et al., 2011), including important woody species, ornamentals, and agricultural crops, such as eggplant, oilseed rape, and cotton. Symptoms associated with Verticillium wilt are stunting, chlorosis, wilting, vascular discoloration, and early senescence (Deketelaere et al., 2017). Verticillium wilt is associated with losses in crop production worldwide, which exceed billions of dollars annually. Verticillium wilt of cotton was first reported in 1914 (Miao et al., 2019), it spread around the world and was introduced to China *via* cotton cultivars from the United States in 1935 (Cai et al., 2009). Since then, Verticillium wilt of cotton has become a devastating fungal disease in cotton planting areas (Pegg et al., 2002). Generally, the occurrence of Verticillium wilt reduces the yield by 10–30%, and in serious cases, the yield can be reduced by more than 80% (Wei et al., 2015). The acreage of Verticillium wilt-infected cotton fields in China is around 2.5 million hectares annually, causing direct economic losses of ca. 250–310 million US dollars (Li et al., 2015).

Recently, several studies were conducted to examine the physiological and molecular mechanisms of plant resistance to *V. dahliae*. Faced with Verticillium wilt, plants can develop resistance through a variety of mechanisms, including cell wall modifications, extracellular enzymes, pattern recognition receptors, transcription factors (TFs), and signal transduction pathways related to salicylic acid (SA), jasmonic acid (JA), or ethylene (ET) (Song et al., 2020). More importantly, the many resistance-related genes and long non-coding RNAs identified and experimentally validated to be involved provide a theoretical foundation for a better understanding of the molecular genetic mechanisms underlying plant resistance to Verticillium. *GhMYB36* encodes an R2R3-type MYB protein in *Gossupium hirsutum* (*G. hirsutum*) and is involved in drought tolerance and Verticillium wilt resistance in *Arabidopsis* and cotton by enhancing the expression of resistance genes (Liu et al., 2022). In addition, *GhWAK7A*, a wall-associated kinase, serves as an important component in cotton’s response to fungal wilt pathogens by complexing with the chitin receptors to positively regulate cotton’s response to *V. dahliae* infections (Wang P. et al., 2020). Long non-coding RNA lncRNA7 and its regulating gene Pectin methylesterase inhibitor 13 (*GbPMEI13*) positively regulate disease resistance via a silencing approach. In contrast, lncRNA2 and its regulating gene Polygalacturonase 12 (*GbPG12*) negatively regulated resistance to *V. dahliae* (Zhang L. et al., 2022). By identifying 41 conserved ENODLs in *G. hirsutum*, Zhang M. et al. (2022) found *GhENODL6* was upregulated under *V. dahliae* stress and hormonal signal and displayed higher transcript levels in resistant cotton than the susceptible. In addition, pectin lyase enhances cotton resistance to Verticillium wilt by inducing cell apoptosis in *V. dahliae* (Zhang et al., 2021). Apart from these, several genes, including *GhLAC15* (Zhang et al., 2019), *GhWAKL* (Feng et al., 2021), *GbCYP86A1* (Wang G. et al., 2020), etc., have been reported to be involved in defense mechanisms against *V. dahliae*.

*G. hirsutum* and *Gossypium barbadense* (*G. barbadense*) are two kinds of widely cultivated allotetraploid cotton species that originated from a hybridization event between *G. arboreum* and *G. raimondii* about 1–2 million years ago (Paterson et al., 2009). *G. hirsutum* and *G. barbadense* have a common allotetraploid ancestor, but the independent domestication process resulted in huge differences in plant type, yield, fiber quality, environmental adaptability, etc. Our previous report demonstrated that *G. hirsutum* was more tolerant to abiotic stress in general than *G. barbadense* (Hu et al., 2019). Even so, in terms of disease resistance, such as Verticillium wilt and Fusarium wilt, *G. barbadense* cultivars show superior performance. Thus, a proposed approach to improve the disease resistance of *G. hirsutum* is to transfer the superior related traits from *G. barbadense* into *G. hirsutum* during the breeding process (Ma et al., 2021). Due to the vitality of resting spores and the overwintering structures produced by microsclerotia, it is difficult to eradicate *V. dahliae* from infected fields. Planting varieties resistant to Verticillium wilt is the most effective way to control the disease. However, the reasons for the formation of the divergence in Verticillium wilt resistance between *G. barbadense* and *G. hirsutum*, as well as its molecular mechanism, are still largely unknown.

Due to the lack of disease-resistant germplasm resources in *G. hirsutum* and the variation in *V. dahliae* strains, the breeding progress for Verticillium wilt resistance seems to be at a standstill. Yield traits and disease resistance are not always able to aggregate well, which brings many barriers for high-yield and high-resistance cotton breeding. Therefore, analyzing the formation of Verticillium wilt resistance differentiation between resistant cultivars and sensitive varieties helps find the core genes of the pathogen response and provides theoretical support for disease-resistant and high-yield breeding not only for cotton but also for more varieties of plants that suffer from Verticillium wilt. In this study, we performed a comprehensive transcriptome analysis of Hai7124 (*G. barbadense*; *V. dahliae*-resistant) and TM-1 (*G. hirsutum*; lower *V. dahliae* resistance) multiple hours after *V. dahliae* infection to identify common and different gene expression and regulation networks, aiming to gain new insight into the divergence of Verticillium wilt resistance between *G. barbadense* and *G. hirsutum* and generate a substantial understanding of host-pathogen interactions and the defense mechanisms in plants.

## Materials and methods

### Plant materials and *Verticillium dahlia* inoculation

Two cotton species, Hai7124 (*G. barbadense*) and TM-1 (*G. hirsutum*), were used in present study. Seedlings were grown in a controlled-environment growth room at 22°C ± 2°C with a photoperiod scheme of 16-h light/8-h dark. After 14 days sowing, we divided these cotton seedings into two identical groups, one inoculated with a *V. dahliae* strain Vd991 spore suspensions (10^7^ conidia/ml in sterile distilled water), the other was treated with equal volume of water as a negative control (mock). The mix of leaves, roots and stems of Verticillium wilt group and mock group’s samples at 0, 12, 24, 48, 72, 96, 120 and 144 hpi were collected respectively. Three biological replicates were performed for each cultivar at each time under fungal and water inoculated treatments. All these samples were instantly frozen in liquid nitrogen and stored at -80°C.

### Transcriptome profiling

Total RNA was extracted using the RNeasy plant mini kit (BSC65S1, BioFlux) along with DNase treatment, and then high-throughput sequencing platform was adopted using the Illumina HiSeq 4000 platform for RNA sequencing. A total of 90 libraries were constructed for Hai7124 and TM-1 (7 time points of 2 cultivars in 3 biological replicates for *V. dahliae* group and 8 time points of 2 cultivars in 3 biological replicates for mock group). The clean reads were generated by processing the raw data whose format was FASTQ to fastp (Chen et al., 2018). Then all the clean reads were aligned to the TM-1 genome (v2.1) (Hu et al., 2019) utilizing hisat2 version 2.1.0 (Kim et al., 2015). The fragments per kilobase of transcript per million mapped reads (FPKM) was calculated to represent gene expression levels (Trapnell et al., 2010).

We performed principal component analysis (PCA) with the R package models^1^ and conducted determination of Pearson correlation coefficients (Benesty et al., 2009) with R version 4.1.2 to investigate similarity among three replicates. Afterwards, RNA differentially expression analysis was carried out, genes induced or repressed of *V. dahliae* inoculation when comparing to mock at the same time point in Hai7124 and TM-1 were identified using the R package DESeq2 (Love et al., 2014) thus leading to seven groups of differentially expressed genes (DEGs) for each cultivar. Only significantly changed genes with an adjusted *p*-value < 0.05 and | log2(fold-change)| > 2 were considered to be differentially expressed genes (DEGs). In addition, Venn diagrams were generated using these DEGs via TBtools version 1.0986853 (Chen et al., 2020). Gene Ontology (GO) enrichment analysis and Kyoto Encyclopedia of Genes and Genomes (KEGG) analysis^2^ were performed. The terms and pathways with a corrected *P*-value < 0.05 were selected for further analysis.

### Identification of nucleotide-binding leucine-rich repeat genes

We defined as nucleotide-binding leucine-rich repeat (NLR) genes that contained at least a nucleotide-binding (Pruitt et al., 2021), a Toll/interleukin-1 receptor (TIR) or a coiled-coil (CC) domain, Leucine-rich repeat (LRR) domain as previous research reported (Van de Weyer et al., 2019). The Hidden Markov Model (HMM) profile of the NB (Pfam accession PF00931), TIR (PF01582), CC (PF05659), LRR (PF00560, PF07725, PF13306, and PF13855) domains was obtained from Pfam website,^3^ and was employed as a query to identify all possible NLR genes using HMMER (V3.0) software (Finn et al., 2011) in Hai7124 and TM-1 genome (Hu et al., 2019) respectively. Eventually, the BLAST search program was used to undermine the coordination between cotton specials to make a convenient comparison in present study.

### Co-expression network construction and hub genes selection

To assess similarities and differences in expression patterns along with *V. dahliae* colonization between Hai7124 and TM-1, the FPKM of the unique DEGs from these two cultivars was used to construct the co-expression networks by way of the R package WGCNA (weighted gene co-expression network analysis) (Langfelder and Horvath, 2008). Adjacency matrices were constructed using the soft threshold power, then networks were identified using a dynamic tree-cut algorithm with a minimum cluster size of 30 and merging threshold of 0.25. The co-expression modules were determined with the default settings and then assigned by a unique color. Hub genes were selected based on their module membership (KME) values in the highest coefficient modules (Langfelder et al., 2013). Cytoscape v3.9.0^4^ were explored to visualize the interaction network of hub genes.

## Results

### Phenotype comparison and sequencing upon *Verticillium dahliae* stress between *Gossypium barbadense* and *G. hirsitum*

**FIGURE 1:**
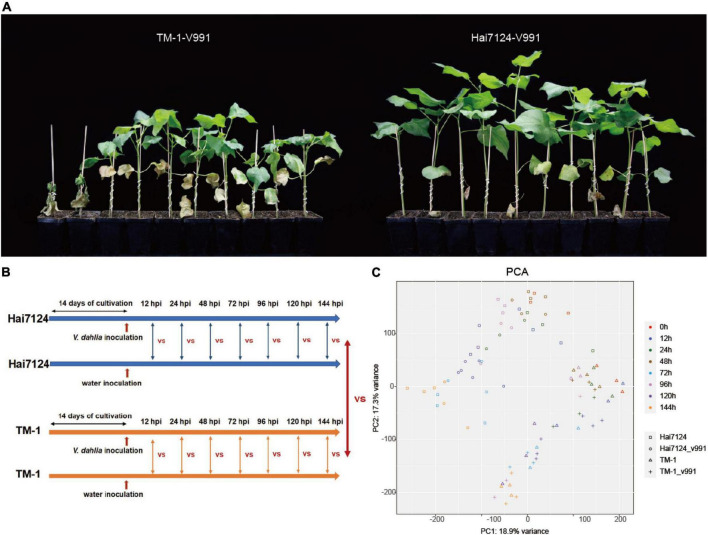
*Gossypium barbadense* (*G. barbadense*) cultivar Hai124 is more resistant to Verticillium wilt than *Gossupium hirsutum* (*G. hirsutum*) TM-1. **(A)** *G. barbadense* cv. Hai124 and *G. hirsutum* acc. TM-1 were used in this experiment. Disease symptoms of cotton plants inoculated with *V. dahliae* for 50 days. **(B)** Seedlings were incubated in growth chambers at 22°C under a 16–8 h day-night period for about 2 weeks (until they grew two true leaves) and then treated with *V. dahliae* and water, and harvested at 0 h, 12 hpi, 24 hpi, 72 hpi, 96 hpi and 144 hpi. **(C)** PCA depicting pairwise Pearson correlation of gene expression values of all samples.

As a generally acknowledged truth for cotton breeders in practice, *G. barbadense* cultivars show significantly higher resistance to Verticillium wilt than *G. hirsutum*. Using the cultivars Hai7124 (*G. barbadense*) and TM-1 (*G. hirsutum*), we performed a disease resistance investigation in the greenhouse to make further comparisons of their resistance differences. The symptoms resulting from *V. dahliae* (Vd991) infection were observed for Hai7124 and TM-1 in the greenhouse. The wilt leaves and chlorosis in TM-1 were much more severe than that in Hai7124 at 50 days post-inoculation (Figure 1A), indicating Hai7124 was more tolerant to Verticillium wilt than TM-1, consistent with previous reports.

Based on the distinct resistance to Verticillium wilt between Hai7124 and TM-1, a mixture of leaves, roots, and stems were collected from Hai7124 and TM-1 after *V. dahliae* inoculation at 7 time points and were subject to RNA sequencing as shown in Figure 1B. A total of 90 libraries were constructed from Hai7124 and TM-1 and transcriptome sequencing was conducted, generating approximately 2.23 million raw reads and 2.21 million clean reads per library. The average Q20 and Q30 were 97.21 and 93.25%, respectively, and the GC content was 43% (Supplementary Table 1). The PCA analysis showed that the three replicates of each sampling group clustered well together, with a correlation variance value of 18.9 and 17.3% (Figure 1C), suggesting the reliability of these data. The RNA-seq data were mapped to the corresponding reference genome and normalized to the FPKM values to quantify the transcript expression. Finally, a total of 49,739 genes expressed (FPKM > 1) in at least one time point were detected in all samples from all 7 time points (12 hpi, 24 hpi, 48 hpi, 72 hpi, 96 hpi, 120 hpi, and 144 hpi).

### Comparison of pathogen-induced expression profiles in Hai7124 and TM-1

**FIGURE 2:**
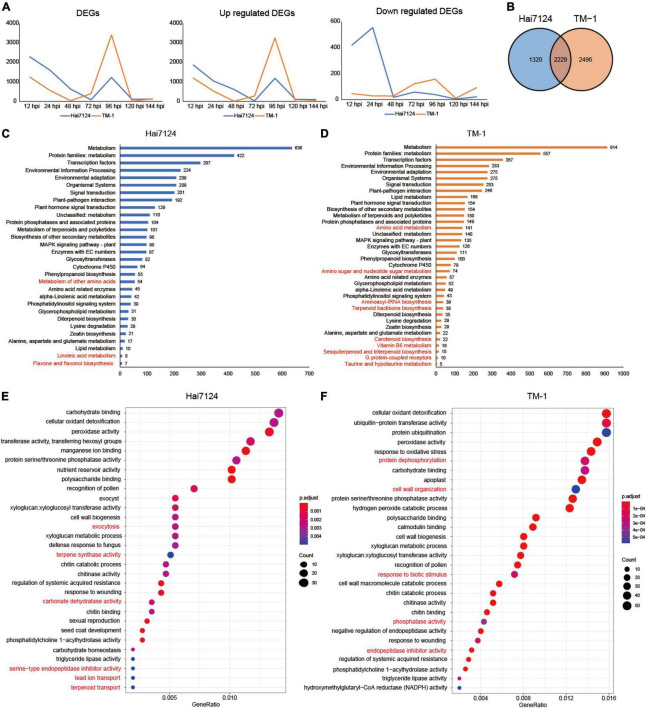
Homoeologous expression bias difference between Hai7124 and TM-1. **(A)** Line chart of DEGs, upregulated and downregulated genes of Hai7124 and TM-1 in each hour post infection. **(B)** Venn diagrams of total DEGs in Hai7124 and TM-1. **(C)** The significant KEGG pathways of the total DEGs identified in Hai7124, the specific pathways were marked in red. **(D)** The significant KEGG pathways of the total DEGs identified in TM-1, the specific pathways were marked in red. **(E)** The top 30 significant GO terms enriched in Hai7124. **(F)** The top 30 significant GO terms enriched in TM-1.

To characterize the gene expression patterns responsive to *V. dahliae* infection in Hai7124 and TM-1, we compared the FPKM of the samples inoculated with *V. dahliae* to mock samples at the same time among the 49,739 expressed genes. Overall, the number of non-redundant genes with an adjusted *p*-value < 0.05 and | log2(fold-change)| > 2 was 3,549 DEGs in Hai7124 and 4,725 DEGs in TM-1 from all 7 time points. In detail, 2,288, 1,593, 631, 85, 1,225, 112, and 117 DEGs were observed at 12, 24, 48, 72, 96, 120, and 144 hpi in Hai7124, respectively, and 1,246, 555, 38, 396, 3,407, 15, and 131 DEGs were identified in TM-1 at 12, 24, 48, 72, 96, 120, and 144 hpi, respectively. The largest number of DEGs (3,407 DEGs) in TM-1 induced by *V. dahliae* infection occurred remarkably later than that of Hai7124 (2,288 DEGs). The former was at 24 hpi and the latter was at 12 hpi. The quantity and time change tendency of the DEGs revealed that the patterns of these two cultivars responding to *V. dahliae* attacks were similar from the aspect of the whole pathogen resistance phase but differed for the small stages (Figure 2A). The total number of DEGs at every time point for Hai7124 and TM-1 immediately increased within 12 hpi (Hai7124: 2288 vs. TM-1:1246 DEGs), reaching the first peak, and after a few hours of decline, the second peak was at 96 hpi (Hai7124: 1225 vs. TM-1:3407 DEGs). Then, there was a swift decrease, indicating that the temporal trends of the DEGs were accordant that both reached their peaks at 12 hpi and 96 hpi, which inferred that these two time points were the key process of the innate immune responses. Furthermore, the number of corresponding DEGs for Hai7124 in the early stage (2,288 at 12 hpi and 1,246 at 24 hpi) of infection far exceeded that of TM-1 (1,593 at 12 hpi and 555 at 24 hpi), elucidating that Hai124 initiated a larger-scale disease resistance and immune response earlier than TM-1. Moreover, the tendency of the up and downregulated DEGs indicated a distinct divergence (Figure 2A). The trend of the upregulated DEGs was consistent with the whole set of DEGs, showing that both varieties had a large number of upregulated genes at the first time point (1869 DEGs in Hai7124 and 1,200 DEGs in TM-1 at 12 hpi). Then, there was a gradual decrease in the upregulated genes, with 1,039 genes at 24 hpi and 613 genes at 48 hpi for Hai7124 and 526 DEGs and 10 DEGs at 24 hpi and 48 hpi for TM-1. Eventually, the upregulated genes for both Hai7124 and TM-1 peaked at 96 hpi (Hai7124: 1186 vs. TM-1: 3250 DEGs) and dropped back to the near-zero level in the following 24 h. However, the number of downregulated genes at 12 hpi and 24 hpi for Hai7124 (419 and 554 DEGs respectively) was distinct from that of TM-1 (46 and 29 DEGs). Consequently, Hai7124 adjusted its gene expression pattern by up or downregulating more rapidly than TM-1 during the first stages of *V. dahliae* infection and carried out an effective tug-of-war with Verticillium wilt, laying the defense mechanisms for disease resistance differentiation.

Throughout the response process of the pathogen, among the DEGs, 1320 (21.84%) in Hai7124 and 2,496 (36.87%) in TM-1 were unique, and 2,229 DEGs (41.29%) were shared (Figure 2B), showing that similar and distinct wilt response mechanisms were present. Furthermore, functional annotation of DEGs for each cultivar was conducted based on the KEGG database and GO enrichment. The 3,549 DEGs identified in Hai7124 were significantly enriched in 30 pathways (Figure 2C), while the 4,725 DEGs from TM-1 were enriched in 36 pathways (Figure 2D). Comparing the KEGG enrichment results of these two varieties, 27 biological pathways were identical, including metabolism, TFs, plant-pathogen interaction, plant hormone signal transduction, metabolism of terpenoids and polyketides, protein phosphatases, etc. Illuminating the DEGs involved in these shared pathways may uncover the mechanism of the early stages following the invasion. However, some pathways were interspecies-specific. Linoleic acid metabolism, flavone and flavonol biosynthesis, and metabolism of other amino acids were solely enriched in Hai7124; while, carotenoid biosynthesis, Vitamin B6 metabolism, sesquiterpenoid and triterpenoid biosynthesis, amino sugar and nucleotide sugar metabolism were markedly enriched in TM-1.

The DEGs in Hai7124 were significantly enriched for 59 GO terms, and there were less than 82 GO terms for TM-1 (Supplementary Table 2). Among the 59 GO terms for Hai7124, the DEGs participating in ubiquitin-protein transferase activity (38) were the largest. Protein ubiquitination, carbohydrate binding, and cellular oxidant detoxification ranked closely in gene number. The defense response by callose deposition in the cell wall had the least number of genes associated with it. As for the 82 terms from TM-1, cellular oxidant detoxification, ubiquitin-protein transferase activity, protein ubiquitination, and cell wall all had 55 genes in each category. Carbohydrate homeostasis ranked last by gene number. A comparison of all the GO categories for each cultivar revealed that 43 GO terms overlapped between Hai7124 and TM-1, 16 specific genes in Hai7124 and 39 in TM-1. The top 30 GO terms enriched were focused (Figures 2E,F). The biological processes involved in signal transduction, exocytosis, terpene synthase activity, carbonate dehydratase activity, lead ion transport, reactive oxygen species metabolic process, magnesium ion binding, and serine-type endopeptidase inhibitor activity were only enriched in Hai7124, while cell wall, hydrolase activity, protein dephosphorylation, magnesium ion binding, lipid transport, and cellulose microfibril organization were solely showed up in TM-1. Notably, the 43 overlapping terms included carbohydrate binding, cellular oxidant detoxification, peroxidase activity, protein serine/threonine phosphatase activity, polysaccharide binding, transferring hexosyl groups, manganese/calmodulin ion binding, cell wall biogenesis, etc.

In summary, the gene expression patterns of Hai7124 and TM-1 in response to *V. dahliae* showed an identical trend in general, but there were large differences in the subtleties. For the quantity and tendency of DEGs by number, they were similar from the aspect of the whole pathogen resistance phase, but the gap in the number of DEGs was quite large at the common key peak time point for Hai124 and TM-1. Moreover, for the enrichment analysis results based on DEGs, Hai7124 and TM-1 showed roughly similar resistance response processes, including protein ubiquitination and cellular oxidant detoxification, in response to the *V. dahliae* infection, but inter-species biases in the extent and intensity of these identical‘ or unique biological processes existed. There were different focuses and distinct mechanisms among them. For instance, the DEGs in Hai7124 played roles in the signal transduction process and exocytosis to activate the wide response, while TM-1 focused on physical defense by utilizing genes that take part in cell wall construction, such as hydrolase activity and cellulose microfibril organization, with 91 DEGs associated with the cell wall in TM-1, whereas there were only 26 in Hai7124.

### Inequivalent distribution of plant disease resistance bioprocesses resulted in the divergence of Verticillium wilt tolerance between *Gossypium barbadense* and *Gossupium hirsutum*

**FIGURE 3:**
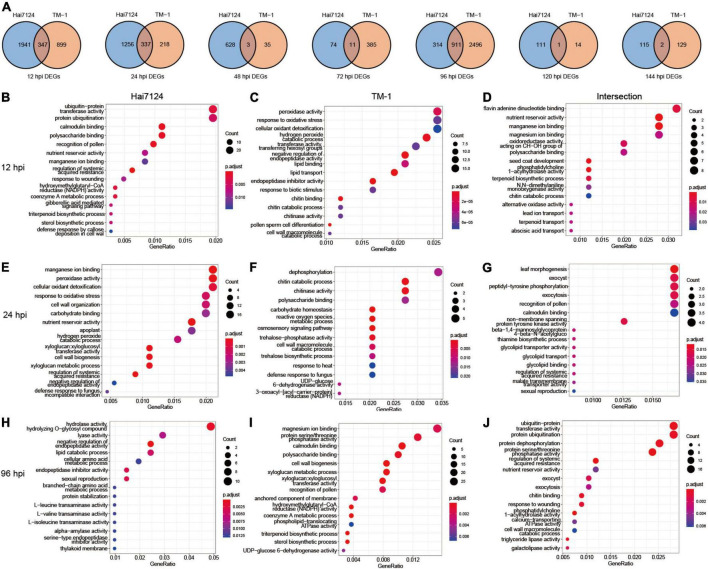
Dynamic gene expression changes during Verticillium wilt infection process. **(A)** Venn diagrams of DEGs with interspecies expression bias at each time point. **(B)** The top 15 significant GO terms enriched in DEGs uniquely expressed in Hai7124 at 12 hpi; **(C)** The top 15 significant GO terms enriched in DEGs uniquely expressed in TM-1 at 12 hpi. **(D)** The top 15 significant GO terms enriched in DEGs commonly expressed in Hai7124 and TM-1 at 12 hpi. **(E)** The top 15 significant GO terms enriched in DEGs uniquely expressed in Hai7124 at 24 hpi; **(F)** The top 15 significant GO terms enriched in DEGs uniquely expressed in TM-1 at 24 hpi. **(G)** The top 15 significant GO terms enriched in DEGs commonly expressed in Hai7124 and TM-1 at 24 hpi. **(H)** The top 15 significant GO terms enriched in DEGs uniquely expressed in Hai7124 at 96 hpi; **(I)** The top 15 significant GO terms enriched in DEGs uniquely expressed in TM-1 at 96 hpi. **(J)** The top 15 significant GO terms enriched in DEGs commonly expressed in Hai7124 and TM-1 at 96 hpi.

To discover inter- and intra-specific expression biases, we compared the DEGs from Hai7124 and TM-1 at each time point in detail. At 96 hpi, 911 DEGs were identified, accounting for the highest number of shared genes, and 347 and 337 DEGs were found at 12 hpi and 24 hpi, respectively. Only 17 DEGs at 48, 72, 120, and 144 hpi were shared in both cultivars. Among them, 3 genes at 48 hpi were found, including a cGMP-dependent protein kinase (*GH_A07G1749*), a Germin-like protein subfamily 1 member (*GH_D11G2744*), and an unknown gene (*GH_D05G3868*). There were 11 genes at 72 hpi, including 4 F-box protein (*GH_A06G2344*, *GH_A06G2345*, *GH_D06G2376*, and *GH_D06G2377*), 2 genes with unknown functions (*GH_A13G0280* and *GH_D10G1713*), an ethylene-responsive transcription factor (ERF) gene *GH_D11G0433*, a basic chitinase named *GH_A06G0558*, a Glutamine synthetase nodule isozyme *GH_A11G1761*, a Glucan endo-1,3-beta-glucosidase *(GH_D09G1316)*, and a nodulin MtN21/EamA-like transporter family gene (*GH_D06G0451*). Moreover, only one gene was in common at 120 hpi encoding the protein plant natriuretic peptide A (*GH_A08G0955)*. Finally, two genes, ethylene-responsive transcription factor (*GH_D04G1148)* and protein ASPARTIC protease in guard cell 2 *(GH_D05G0626)*, were prominent at 144 hpi (Figure 3A).

Subsequently, we paid more attention to the annotation and enrichment terms of the DEGs at 12 hpi, 24 hpi, and 96 hpi (Figures 3B–J and Supplementary Table 3). First, there were 1,941 DEGs specifically expressed in Hai7124 at 12 hpi, which were concentrated in diverse biological processes, including protein ubiquitination, calmodulin binding, polysaccharide binding, nutrient reservoir activity, manganese ion binding, regulation of systemic acquired resistance, xyloglucan: xyloglucosyl transferase activity, response to wounding, hydroxymethylglutaryl-CoA reductase (HMGR) activity, coenzyme A metabolic process, gibberellic acid (GA) mediated signaling pathway, sterol biosynthetic process, and defense response by callose deposition in the cell wall (Figure 3B). Among these, genes related to protein ubiquitination, which is one of the major pathways of protein degradation, were dominant, including *GhPUB22* and *GhPUB23*, E3 ubiquitin-protein ligase, confirming their role in the resistance to *F. oxysporum* infection in cotton (Chen et al., 2014). Additionally, *GH_D02G1964*, which encodes E3 ubiquitin-protein ligase RMA1H1 (RING membrane-anchor 1), shows strongly enhanced tolerance to drought stress (Lee et al., 2009; Chen et al., 2021). Also, xyloglucosyl transferase encodes an enzyme involved in cell wall elongation and restructuring (Cosgrove, 2005). However, only 899 DEGs were particularly expressed in TM-1 during the same period (12 hpi), which was significantly less than Hai7124 (1941 DEGs). Those genes’ functions were mainly concentrated in several processes, such as peroxidase activity, response to oxidative stress, cellular oxidant detoxification, hydrogen peroxide catabolic process, transferase activity: transferring hexosyl groups, and negative regulation of endopeptidase activity (Figure 3C). Most of these were related to scavenging reactive oxygen species (ROS), which is involved in the plant immune system (Gao et al., 2021; Wang et al., 2022). The 2 cultivars showed a similar expression pattern for 347 genes jointly at 12 hpi, and the biological response processes were flavin adenine dinucleotide binding (Li et al., 2021), nutrient reservoir activity, manganese ion and magnesium ion binding, and oxidoreductase activity (Figure 3D). Thus, we inferred that the intracellular redox reactions were dominant in TM-1 at 12 hpi, while Hai7124 developed a wide variety of anti-disease defense responses to better prepared molecularly to deploy critical defense response proteins.

Second, at 24 hpi, 1256 specifically expressed genes in Hai7124 were dominant. The top GO categories were manganese ion binding, reactions related to oxidative stress, cell wall organization/biogenesis, carbohydrate binding, nutrient reservoir activity, and xyloglucan: xyloglucosyl transferase activity (Figure 3E). Simultaneously, the Hai7124-specific-DEGs acted on the response of intracellular oxidative stress and the reconstruction of the cell wall. The functions of 218 DEGs unique to TM-1 converged on dephosphorylation, chitin catabolic process/chitinase activity, polysaccharide binding, carbohydrate homeostasis, ROS metabolic process, etc. (Figure 3F). Considering that the addition or removal of phosphate groups (dephosphorylation) acts as a biological “on/off” for many reactions (Ke et al., 2009; Shi, 2009), TM-1 may have initiated certain anti-disease responses. Moreover, 337 DEGs overlapped between Hai7124 and TM-1 at 24 hpi, and the main biological processes represented by these genes were leaf morphogenesis, peptidyl-tyrosine phosphorylation, exocytosis, and non-membrane spanning protein tyrosine kinase activity (Figure 3G). Thus, the biological functions of the DEGs shared by the two cultivars were relatively scattered. The reactions of oxidative burst were continuous between Hai7124 and TM-1 in the early stage of pathogen invasion (0–24 hpi). However, the intensity and extent of this reaction varied greatly between the two species based on the DEGs numbers involved.

At the 96 hpi, the GO processes revealed 314 DEGs unique in Hai7124 that were classified as hydrolase activity/hydrolyzing O-glycosyl compound, lyase activity, negative regulation of endopeptidase activity, lipid catabolic process, etc. (Figure 3H). In contrast, the enriched terms for TM-1 revealed 2,496 species-specifically expressed genes that were similar with the specific GO terms of Hai7124 at 12 hpi, including magnesium ion binding, protein serine/threonine phosphatase activity, calmodulin binding, polysaccharide binding, cell wall biogenesis, cell wall biogenesis, anchored component of membrane, HMGR activity, coenzyme A metabolic process, phospholipid-translocating ATPase activity, triterpenoid biosynthetic process, and sterol biosynthetic process (Figure 3I). Importantly, the shared 911 DEGs at 96 hpi enriched as protein ubiquitination, protein dephosphorylation, protein serine/threonine phosphatase activity, and regulation of systemic acquired resistance (Figure 3J). The DEG enrichment analysis of these two species at 96 hpi hinted that the disease resistance defense response initiated by TM-1 had an explosive increase, and TM-1 then increased to “catch up” with the resistant type Hai7124 only at 96 hpi, suggesting a delayed recognition and response to *V. dahliae* compared to Hai7124.

In summary, when Verticillium wilt began to invade the cotton plants at 12, 24, and 96 hpi, *G. barbadense* Hai7124 and *G. hirsutum* TM-1 activated corresponding defense measures that shared both commonalities and showed significant interspecific differences. Particularly, Hai7124 developed a broader resistance response at 12 hpi to trigger an effort to contain the invading pathogen, which included xyloglucosyl transferase activity, HMGR activity, coenzyme A metabolic process, GA-mediated signaling pathway, sterol biosynthetic process, and callose deposition in the cell wall. While TM-1 focused on regulating the redox status of the plant cells and protecting them from the oxidative burst and generated ROS in the early stages. It was not until 96 hpi (later stages) that TM-1 activated intensive resistance responses. These results suggested the inequivalent distribution of plant disease resistance bioprocesses activated by DEGs may gradually result in the divergence of Verticillium wilt tolerance between *G. barbadense* and *G. hirsutum*.

### *Gossypium barbadense* was superior to *Gossupium hirsutum* in terms of the number and expression level of resistance-related genes

**FIGURE 4:**
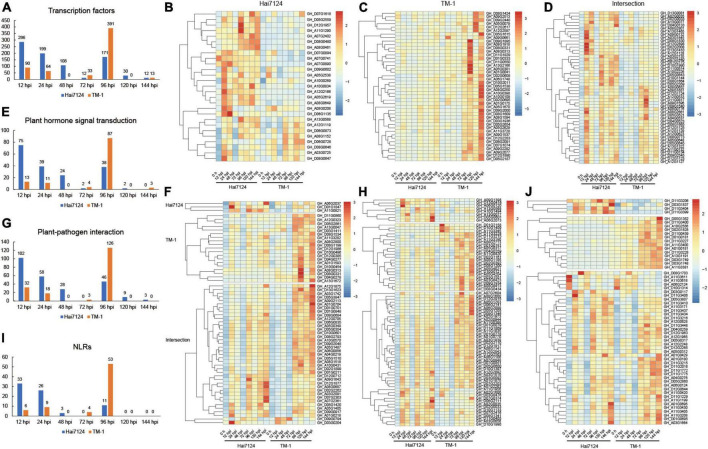
The quantitative and expression level relationship of the four categories’ genes with time changes. **(A)** Number of DEGs in transcription factors with infection time. **(B)** Heatmap of gene expression patterns of TFs uniquely expressed in Hai7124. **(C)** Heatmap of gene expression patterns of TFs uniquely expressed in TM-1. **(D)** Heatmap of gene expression patterns of TFs commonly expressed in Hai7124 and TM-1. **(E)** Variation of the number of DEGs in plant hormone signal transduction with infection time. **(F)** Heatmap of gene expression patterns of DEGs in plant hormone signal transduction. **(G)** Variation of the number of DEGs in plant-pathogen interaction with infection time. **(H)** Heatmap of gene expression patterns of DEGs in plant-pathogen interaction. **(I)** Variation of the number of DEGs in NLRs with infection time. **(J)** Heatmap of gene expression patterns of DEGs in NLRs.

A large number of studies have confirmed that transcription factors (Wang et al., 2018; Wani et al., 2021), plant hormone signal transduction (Verma et al., 2016; Zhou and Zhang, 2020), plant-pathogen interaction (Dodds and Rathjen, 2010; Han, 2019), and NLRs genes (Yuan et al., 2021a; Zhai et al., 2022) are involved in plant disease resistance and immune processes. Thus, we focused on these targets during the time course of our experiment to reveal the difference in resistance between *G. barbadense* and *G. hirsutum*. We defined TF DEGs throughout the whole defense stage (3549 in Hai7124 and 4,725 in TM-1), and 616 unique TFs were identified in total (396 in Hai7124 vs. 476 in TM-1). Among these, 256 TFs were shared by the two species. For the 396 TFs in Hai7124, they consisted of 148 AP2/ERFs, 63 WRKYs, 34 MYBs, 21 MYB-related, 27 bHLHs, 18 GRASs, 13 NACs, 8 HSFs, 7 bZIPs, 7 GATAs, 5 LBDs, 4 SBPs, 3 Trihelixs, and 2 C2H2s. The TF number showed a zigzag trend from the time of the invasion of *V. dahliae* in Hai7124 that started at its highest level of 286 TFs at 12 hpi, then decreased to 12 TFs at 72 hpi and followed another small climax at 96 hpi with 171 TFs, and then decreased rapidly to a low level (Figure 4A). On the other hand, a total of 476 TFs were found in TM-1, including 139 AP2/ERFs, 91 WRKYs, 48 MYBs, 18 MYB-related, 41 bHLHs, 13 GRASs, 15 NACs, 15 HSFs, 14 bZIPs, 9 GATAs, 4 LBDs, 2 SBPs, 7 Trihelixs, and 2 C2H2s. While the number of TFs in TM-1 presented a zigzag shape, it was relatively stable at the early stage (90, 64, 0, and 33 TFs at 12, 24, 48, and 72 hpi, respectively) but fluctuated extremely in the later stage, showing 391 TFs were involved in disease resistance at 96 hpi, which was significantly more than that in Hai7124. In addition, the expression levels of these TFs were uniquely expressed or co-expressed between Hai7124 and TM-1 (Supplementary Figure 1) but all responded to *V. dahliae* infection, such as *GhMYB36*, which is reported to encode an R2R3-type MYB protein that is involved in drought tolerance and Verticillium wilt resistance in Arabidopsis and cotton by enhancing the expression of resistance genes (Liu et al., 2022). Its expression level increased dramatically at 12 hpi in Hai7124 and TM-1. For those TFs specifically expressed in *G. barbadense*, these genes’ expression levels were significantly increased upon *V. dahliae* infection (12 hpi), including *GH_A07G0890* (NAC), which had been verified to function in relation to stress-induced fruit ripening in banana (Yan et al., 2021), *GH_D06G1135*, which is a WRKY family transcription factor that serves as protein in polar localization during asymmetric division and redistribution, *GH_A12G2166* and *GH_A03G0725* (ERF), which are members of the ethylene-responsive transcription factors (Figure 4B). On the contrary, most of the TFs uniquely in TM-1 responded to Verticillium wilt at 96 hpi or even later, including *GH_D09G1895* (bHLH), *GH_A01G0041* (bZIP), and *GH_A08G0250* (MYB) (Figure 4C). Moreover, the shared TFs showed a uniform trend that the expression levels in Hai7124 were relatively high at 12 hpi and 24 hpi, but the significant increase in expression level did not show up until 72 hpi in TM-1 for the same TF (Figure 4D). Among them, *GH_A08G0652*, *GH_A12G1128*, and *GH_A08G1651*, the ethylene-responsive transcription factors of the AP2/ERF family are involved in a wide range of stress tolerance activities and form an interconnected stress regulatory network (Xie et al., 2019).

Moreover, 93 and 103 DEGs were shown to play a role in plant hormone signal transduction in Hai7124 and TM-1. Among these, 55 genes were identical in these two cultivars, including those related to jasmonate-zim-domain proteins (JAZ) (20 genes), which are involved in the regulation of the jasmonic acid-mediated signaling pathway, Gibberellin signaling DELLA protein (8 genes), auxin-responsive protein SAUR (7 genes), auxin responsive GH3 gene family (CH3) (4 genes), protein brassinosteroid insensitive 1(BRI1) (4 genes), auxin-responsive protein (IAA) (4 genes), gibberellin receptor (GID1) (3 genes), salicylate activated NPR1, which interacts with TGA transcription factors to induce the expression of a large set of pathogenesis-related proteins (TGA) (2 genes) (Beckers and Spoel, 2006), jasmonic acid-amino synthetase (JAR1) (1 gene), the type-B authentic response regulator (ARR-B) genes involved in cytokinin signaling (1 gene), and ABA responsive element binding factor (ABF) (1 gene). In the first 72 h, the number of DEGs related to plant hormone signal transduction boosted to a maximum at 12 hpi (75 DEGs) and then began to gradually decrease with a small peak at 96 hpi (38 DEGs) and finally decreased to zero in the next 48 h (2 and 0 DEGs at 120 hpi and 144 hpi), indicating that the plant hormone signal transduction process triggered by *V. dahliae* in the *G. barbadense* plants was completed. In contrast, the trend of the quantitative changes of the plant hormone signal transduction genes was completely divergent in TM-1. A small peak was found at 12 hpi and 24 hpi (13 and 11 DEGs) and reached its highest point at 96 hpi (87 DEGs) (Figure 4E). The genes included *GH_D01G1847* (gibberellin receptor GID1), *GH_A01G0218* (jasmonate-zim-domain protein 10), and *GH_A07G2361* (AUX/IAA transcriptional regulator family protein). Instead, for TM-1, the vast majority of genes remained at normal expression levels from 0 to 48 h and did not respond until 72 hpi, as seen for *GH_D09G0917* (TIFY 5A), which serves as JAZ and plays an important role in JA signal transduction (Figure 4F). We inferred that genes related to plant hormone signal transduction showed higher expression levels in Hai7124 than TM-1 at 12 hpi and 24 hpi. The expression of many genes in TM-1 were not changed dramatically until 72 hpi, providing evidence that the plant hormone signal transduction responding to Verticillium wilt was significantly delayed in TM-1 compared to Hai7124.

Furthermore, the quantitative changes of plant-pathogen interaction genes were highly similar to plant hormone signal transduction (Figure 4G). There were 123 and 151 DEGs associated with the plant-pathogen interaction in Hai7124 and TM-1, respectively, and 89 genes appeared in both Hai7124 and TM-1. The expression levels of these genes showed pronounced interspecific differentiation, illustrating that there were two high-expression response time periods for genes related to the plant-pathogen interaction in Hai7124. The first interval was 12 hpi to 24 hpi, and the second was 72 hpi to 144 hpi. However, the high expression interval of such plant-pathogen interacting genes in TM-1 was basically between 72 and 144 hpi (Figure 4H), suggesting that the gene expression taking part in the plant-pathogen interaction in Hai7124 was quicker and stronger than TM-1. Taking *GH_D07G2416* (SAG101) as an example, it encodes a senescence-associated carboxylesterase 101 and provides a major barrier to infection by both host-adapted and non-host pathogens during plant immunity (Lapin et al., 2019; Sun et al., 2021). In Hai7124, its expression level jumped up immediately at 12 hpi, and after a brief down-regulation at 48 hpi, it continued to express highly from 72 to 144 hpi. While in TM-1, it was significantly overexpressed at 96 hpi, with inconspicuous up-regulated expression the rest of the time, which may have a profound impact on plant disease resistance.

Eventually, a total of 744 genes have been identified to possess NBS domains in TM-1, and 748 genes were revealed in Hai7124. The total NLR genes were 428 in Hai7124 and 426 in TM-1, certificated to be irreplaceable in ETI process in plants. However, 45 and 55 NLR genes in Hai7124 and TM-1, respectively, were identified as DEGs, the overlapping part was 41 NLRs. The tendency of NLRs over time was consistent with the changing pattern of DEGs regarding the transcription factors, plant hormone signal transduction, and plant-pathogen interaction. The number of NLR genes corresponding to TM-1 at 12 hpi and 24 hpi were 6 and 9, respectively, and reached 53 NLRs at 96 hpi. While, for Hai7124, 33 and 26 NLR genes were differentially expressed at 12 hpi and 24 hpi, respectively, and there were 11 NLRs at 96 hpi (Figure 4I). Additionally, the expression patterns of these NLR genes in Hai7124 and TM-1 were examined to understand how they adjusted and survived in response to pathogen infection (Figure 4J). Four genes specifically expressed in Hai7124, *GH_D11G3206*, *GH_D03G1837*, *GH_D11G3404*, and *GH_D11G3399*, belonged to TIR-NBS-LRR class. The remaining 41 NLR genes were induced both in Hai7124 and TM-1. Notably, 26 responded to *V. dahliae* invasion quickly and enhanced their expression level at 12 hpi in Hai7124. These began to increase their transcript level at 72 hpi in TM-1, such as *GH_D11G3437* and *GH_D11G3434*. Moreover, the magnitude of the up or down regulation of the DEGs in *G. barbadense* was much larger than in *G. hirsutum*, such as *GH_A11G3611, GH_A11G3618, GH_A06G2124*, and *GH_D10G1314*. Overall, a species-biased expression trend still existed that for most of the NLR genes, the time points of induction were earlier in Hai7124 than TM-1. Considering that the NLR genes are irreplaceable and play an important role in the plant immune system, we inferred that these NLR genes play a predominant part within 48 hpi in dealing with *V. dahliae* attacks and shape the disease resistance quality substantially in Hai7124.

All the above assays confirmed that genes playing irreplaceable roles in disease resistance were postponed in TM-1, regarding expression level and gene number, compared to Hai7124. Hence, we proposed that the first 48 hpi after infecting *V. dahliae* is an essential life-or-death moment to build internal and external walls of defense to resist the invasion of *V. dahliae* in cotton plants.

### Co-expression network in response to Verticillium wilt and hub genes

**FIGURE 5:**
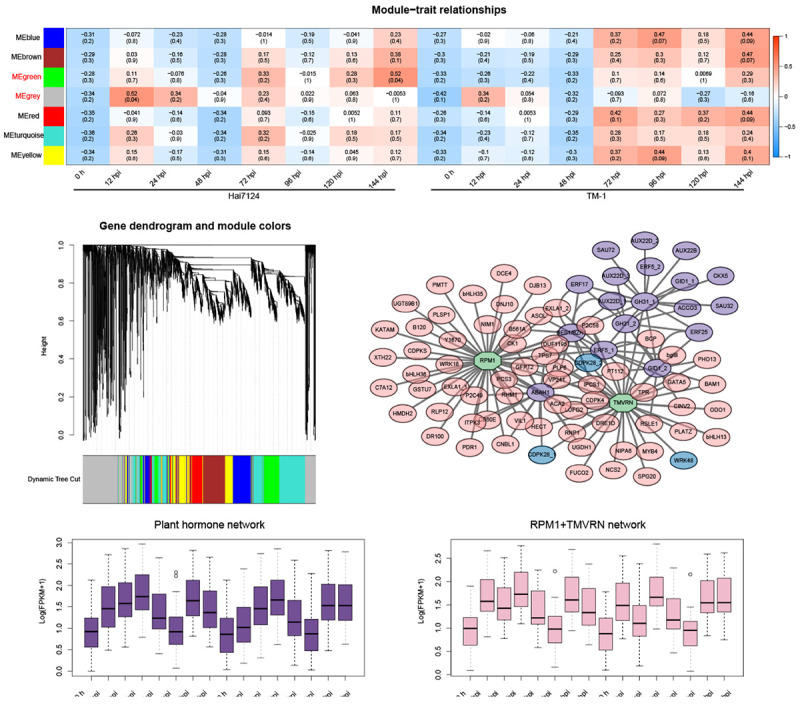
Weighted gene co-expression network analysis (WGCNA). **(A)** WGCNA of DEGs from Hai7124 and TM-1 at each hour post-inoculation. The modules showing a high degree of correlation (*P* < 0.05) with samples are in red. **(B)** Hierarchical cluster tree of WGCNA analysis, the co-expression modules labeled with seven different colors based on the calculation of eigengenes. **(C)** The correlation network of major genes from the green modules. The genes related to phytohormones were in purple, genes relative to plant-pathogen interactions were in blue, genes belonged to NLRs were in green, and hub genes from other categories were in pink. **(D)** Box-plots of hub genes in plant hormone showing expression pattern of the network. **(E)** Box-plots of hub genes in NLRs relative subnetworks showing expression pattern of the network. The *x*-axis indicates time points (0 h, 12 hpi, 24 hpi, 72 hpi, 96 hpi, and 144 hpi), and gene expression data were normalized to Log2(FPKM + 1).

To further understand the relationship between gene regulation and cotton defensive resistance to *V. dahliae*, 6,045 non-redundant DEGs from Hai7124 and TM-1 were subjected to WGCNA, and a co-expression network was generated (Figure 5A and Supplementary Table 4). In total, 7 modules were obtained according to a pairwise correlation analysis based on the gene expression profiles of Hai7124 and TM-1 (Figure 5B). The green module and grey module–trait relationships (indicated with red underlines) were significant (*P*-value < 0.05). The gene expression levels of the green module (393 genes) showed an increase at 12 hpi, 24 hpi, 72 hpi, 96 hpi, and 144 hpi in Hai7124, while in terms of TM-1, the expression level of the genes in the green module did not begin to rise until 72 hpi. Simultaneously, the transcript levels of the grey module genes (886 genes) in Hai7124 were soaring at 12 hpi and 24 hpi. Meanwhile, although the genes in this module were also upregulated at 12 hpi and 24 hpi in TM-1, the intensity was 0.34 and 0.054, respectively, which were far less than that of Hai7124 (0.52 and 0.34), and its expression level stabilized at a relatively low level in the later stage of *V. dahliae* infection.

A total of 70 hub genes related to *V. dahliae* resistance in cotton were identified using the criteria of eigengene connectivity (KME) > 0.98 (Supplementary Table 5). Consistent with the GO and KEGG annotations, the candidate hub genes were involved in biological processes, such as plant hormone signal transduction (CIGR1, JAZ1, B561P), the MAPK signaling pathway [WRK22, Y2451 (LRR)], the plant-pathogen interaction (CML27), xyloglucan galactosyltransferase activity (KATAM), and the heavy metal transport/detoxification process (HMTD).

One core network consisting of three small subnetworks was identified as a key model (green module) of the *V. dahliae* response (Figure 5C). Genes with known functions were selected based on the interaction relationship of two hub genes (RPM1 and TMVRN contained the NBS domain and TIR-NBS-LRR domain, respectively) that belonged to NLRs and plant hormones. This correlation network of the high-weight value genes in this green module was visualized using gene interaction edges with high weights. The interaction network results showed that the disease resistance protein NLR initiated a wide range of downstream reactions to cope with the invasion of foreign pathogens. For example, a large number of phytohormone-related genes were closely associated with these two plant resistance proteins, such as jasmonate-zim-domain protein 10 (JAZ10), ethylene-forming enzyme (ACCO3), cytokinin oxidase 5 (CTX5), brassinosteroid signaling positive regulator family protein (BES1/BZR1), abscisic acid 8′-hydroxylase 1 (ABAH1), and AUX/IAA transcriptional regulator family protein (AUX22B), forming a phytohormone response network. Apart from that, calcium-dependent protein kinase (CDPKS) genes, inositol-tetrakisphosphate kinase (ITPK3), vacuolar protein sorting-associated protein (VP241), rhamnose biosynthesis (RHM1), protein phosphatase 2C family protein (P2C49), and E3 ubiquitin-protein ligase (LOFG2) were activated by RPM1 and TMVRN. This weighted co-expression network revealed that the response mechanism of plant disease resistance proteins reacted to the invasion of exogenous pathogens and the close cooperative relationship between them and plant hormones. Furthermore, box plots of the hub genes in the plant hormone and NLR relative subnetworks showed that the expression pattern of these hub genes had a consistent trend, which ensured the reliability of this network (Figures 5D,E and Supplementary Table 6). It turned out that the correlation of genes in each sub-network was relatively high and clustered well.

## Discussion

### Rapid and high intense pathogen response in the early stages of infection is critical for cotton disease resistance

In general, Verticillium wilt causes great reductions in yield and fiber quality in cotton, and understanding the mechanisms of resistant cotton cultivars underlying cotton *V. dahliae* colonization is critical for mining key genes for breeding disease-resistant and high-yield varieties. In the current study, we observed the disease symptoms after treating with *V. dahliae* and confirmed that the *G. barbadense* Hai7124 showed a stronger tolerance over *G. hirsutum* TM-1 (Figure 1A). Our transcriptome analysis between the two cultivars further clarified the response mechanisms adopted in the resistant cultivar plants. The peak of the DEG number in TM-1 (12 hpi and 24 hpi) induced by *V. dahliae* infection was remarkably later than that of Hai7124 (96 hpi) (Figures 2A, 3A). Specifically, in the early stage of the response to Verticillium wilt (0 h to 48 hpi), the number of DEGs in Hai7124 was strikingly more than TM-1. Furthermore, the Verticillium wilt defense response of *G. barbadense* was preferential to that of *G. hirsutum* in terms of the number and expression level of resistance-related genes, including TFs, plant hormone signal transduction, plant-pathogen interaction, and NLRs genes (Figure 4). The response time and the intensity of expression of these genes in *G. barbadense* Hai7124 were prominent over TM-1. Moreover, the results of WGCNA (Figure 5B) also supported the conclusion that the response time of Verticillium wilt of Hai7124 was earlier than that of TM-1. Tolerant cultivars tend to respond more quickly to a biotic or abiotic stress than susceptible cultivars, such as lentil (*Lens culinaris*) fight against *Ascochyta lentis* infection during the first 24 hpi (Khorramdelazad et al., 2018).

**FIGURE 6:**
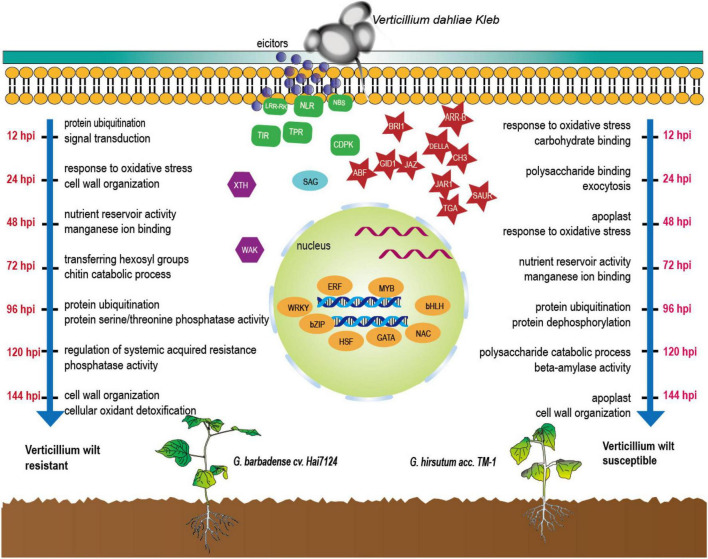
Defense-related molecules involved in response of cotton to *V. dahliae* during the first 144 hpi.

During the infection of the two cultivars by *V. dahliae*, the dominant biological responses and the active molecules were completely different at different times (Figure 6). As for the molecular functions and biological processes in response underlying the initiated DEGs, based on the KEGG and GO annotations, we found that Hai7124 developed a broader resistance response at the early stage of response (0 h to 48 hpi) against Verticillium wilt by up or down regulating a large number of genes, resulting in processes like xyloglucosyl transferase activity, NADPH activity, coenzyme A metabolic process, GA mediated signaling pathway, sterol biosynthetic process, defense response by callose deposition in the cell wall. Xyloglucosyl transferase encodes an enzyme involved in cell wall elongation and restructuring (Cosgrove, 2005), and it can repair and strengthen cell wall defenses; Sterols are involved in plant responses to external stress, and brassinosteroids (BRs) are an important group of plant steroid hormones involved in numerous aspects of plant life including growth, development and response to various stresses (Nolan et al., 2017), thus forming a highly effective defensive response to Verticillium wilt attack in Hai7124. While TM-1 had a smaller amount of DEGs being activated at the first stage, the dominant process at 12 hpi were response to oxidative stress and carbohydrate binding which lacked of direct correlation when compared to protein ubiquitination and signal transduction in Hai7124 at 12 hpi. And it was in the latter stage (after 48 hpi) that TM-1 activated these intensive resistance responses. DEGs associated with protein ubiquitination and protein dephosphorylation which plays key role in plant immune system were increase rapidly (Popovic et al., 2014; Hoang et al., 2019). Moreover, when the genes responded at the same time in Hai7124 and TM-1, the gene expression pattern of *G. barbadense* was often higher than that of *G. hirsutum*, suggesting that some core pathogenic immune-related responses were absent or delayed at critical time points in TM-1. Therefore, we reasonably speculate that missing the prime time for a powerful and extensive defense against foreign pathogenic microorganisms shaped the compromised resistance to *V. dahliae* in *G. hirsutum*. TM-1 compared to *G. barbadense* Hai7124.

### The construction of a finely tuned control resistance system against *Verticillium dahliae*

Although plants are immobile, they still can adjust the expression patterns of certain genes by receiving stimuli signals from the outside world to form a powerful finely tuned defense system to achieve the purpose of resisting the invasion of external pathogens. Faced with *V. dahliae*, cotton can construct that kind of system to protect itself by developing resistance through a variety of mechanisms, including cell wall modifications, extracellular enzymes, plant-pathogen interaction, transcription factors activity, and signal transduction pathways related to plant hormones. However, when it comes to the specific resistance systems based on genes induced by *V. dahliae*, the differentiation among the breeds becomes more pronounced (Figure 6).

We propose a hypothesis that the defense system established by Hai7124 is fast, relatively continuous, extensive, and high-strength, while TM-1 adopts a disease defense model whose core resistance-related processes are delayed. The genes postponed in TM-1 were mainly TFs, enzymes with EC numbers, or those participating in key steps of the plant-pathogen interaction, plant hormone signal transduction, the mitogen-activated protein kinase (MAPK) signaling pathway, membrane trafficking, transporters, and protein phosphatases. All of these are closely linked to the plant immune system against pathogens. Many TF families have been previously reported to be differentially expressed in plants as a reaction to bacterial, fungal, and viral infection (Amorim et al., 2017; Xie et al., 2019; Wani et al., 2021). MAPK cascades play pivotal roles in signaling plant defense against pathogen attack and also can emerge as battlegrounds of plant-pathogen interactions (Meng and Zhang, 2013). Moreover, plant immunity must be tightly controlled to avoid activation of defense mechanisms in the absence of pathogen attack, thus protein phosphorylation is a common mechanism regulating immune signaling (Liu et al., 2020). The addition or removal of phosphate groups (dephosphorylation) acts as a biological “on/off” for many reactions (Ke et al., 2009; Shi, 2009).

Among these genes, the most interesting genes, RPM1 and RPS2, belong to NLRs and are confirmed to recognize pathogen effector perturbations and activate ETI (Kourelis and van der Hoorn, 2018). Coincidently, RPM1 was one of the hub genes in the highly significant module (green) of WGCNA whose annotation is disease resistance RPP13-like protein 4. There were two NLR genes in this module. The other was TMVRN, which contains the TIR-NBS-LRR domain and is described as a disease-resistance protein. In a co-expression sub-network centered on TMVRN, this gene activates functional genes associated with plant stress resistance, such as BCP (Blue-copper-binding protein), GFPT2 (Glutamine–fructose-6-phosphate aminotransferase), IPCS1 (Inositol phosphorylceramide synthase 1), and RSLE1 (Zinc finger BED domain-containing protein RICESLEEPER 1). In addition, the trends in the transcript levels of these network genes activated by TMVRN and RPM1 were consistent with the previously mentioned results that Hai124 responded earlier and stronger to *V. dahliae* than TM-1.

Overall, these results finally showed that Hai714 opened a stronger and more extensive defense system involving TFs, plant hormones, the plant-pathogen interaction, PTI and ETI responses, and other processes effectively preventing *V. dahliae* from further invasion of the cotton. While in terms of TM-1’s delayed resistance system, the pathogenic bacteria received less resistance during the colonization process, resulting in more irreversible physiological and biochemical damage to the cotton plant, which ultimately manifested as the relatively weak resistance to Verticillium wilt. In addition, this phenomenon may indicate that TM-1’s disease resistance was not a typical sensitive type, and the defense was weak in the initial stage such that only small amounts of pathogens were invaded.

## Conclusion

In this study, a comparative transcriptome analysis between *G. barbadense* and *G. hirsutum* was performed to clarify their response mechanisms when Verticillium wilt attacked. Both Hai7124 and TM-1 showed consistent trends of differential gene expression within 12 hpi of *V. dahliae* invasion that was reduced at 48 h and then, increased to a greater extent and intensity at 96 h. While Hai7124 initiated more DEGs at the early response stage over TM-1 and the co-expression of these genes developed a broader resistance response, TM-1 increased to “catch up” with the resistant type only at 96 hpi, suggesting a delayed recognition and response to *V. dahliae* compared to Hai7124. Moreover, for the essential resistance-related genes, including TFs, plant hormone signal transduction, the plant-pathogen interaction, and NLRs, Hai7124 adopted a more immediate, powerful, and extensive response mode to actively fight against the invasion of *V. dahliae* in the critical early stages over TM-1. All these phenomena might contribute to the disease defense mechanisms and lay the foundation for candidate gene investigation in cotton and other plants.

## Data availability statement

The datasets presented in this study can be found in online repositories. The names of the repository/repositories and accession number(s) can be found below: https://www.ncbi.nlm.nih.gov/genbank/, PRJNA839539.

## Author contributions

YH and TZ conceived and designed the experiments. LH and ZH performed the experiments and wrote the manuscript. YZ and ZS performed the experiments and FD analysis of RNA-seq data. JC, SJ, CH, YC, JZ, and BX revised the manuscript. GQ, YWC, LX, and SY performed the experiments. All authors contributed to the article and approved the submitted version.

## Footnotes

1
https://cran.r-project.org/web/packages/gmodels/index.html

2
https://www.genome.jp/kegg/

3
http://pfam.xfam.org/

4
https://cytoscape.org/

## Supplementary material

Supplementary Figure 1 Heatmap of the expression levels of TFs in Hai7124 and TM-1.

Supplementary Table 1 RNA-seq data output quality.

Supplementary Table 2 GO enrichment results of Hai7124 and TM-1.

Supplementary Table 3 GO enrichment results of Hai7124 and TM-1 at 12, 24 and 96 hpi.

Supplementary Table 4 WGCNA information of TM-1 and Hai7124.

Supplementary Table 5 List of the candidate hub genes with module KME values over 0.98.

Supplementary Table 6 Hub genes used for network instruction and their gene annotations.
