# Supplementary figures and images for: Advanced genes expression pattern greatly contributes to divergence in Verticillium wilt resistance between Gossypium barbadense and Gossupium hirsutum
Source: Front Plant Sci. 2022 Aug 1;13:979585. doi: 10.3389/fpls.2022.979585 (PMC9376480; doi:10.3389/fpls.2022.979585)

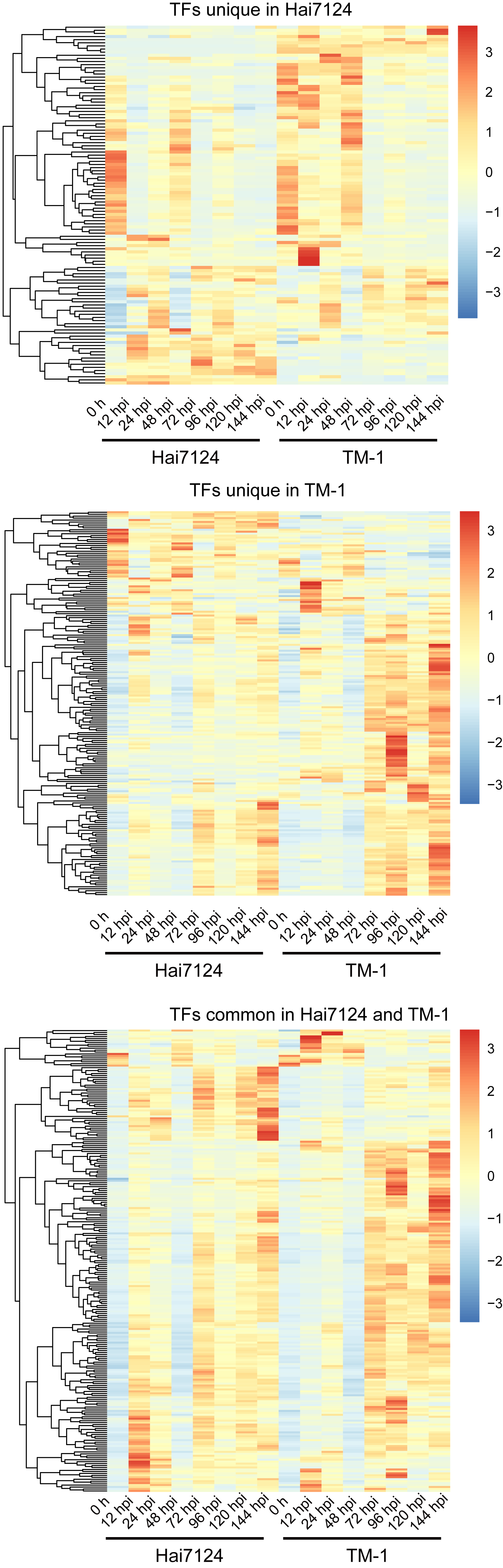

Supplement: Supplementary Figure 1 — Heatmap of the expression levels of TFs in Hai7124 and TM-1. [file Image_1.TIF]
